# Human Liver-Derived Stem Cells Improve Fibrosis and Inflammation Associated with Nonalcoholic Steatohepatitis

**DOI:** 10.1155/2019/6351091

**Published:** 2019-06-02

**Authors:** Stefania Bruno, Maria Beatriz Herrera Sanchez, Chiara Pasquino, Marta Tapparo, Massimo Cedrino, Ciro Tetta, Giovanni Camussi

**Affiliations:** ^**1**^ Department of Medical Sciences, University of Torino, Torino, Italy; ^2^Molecular Biotechnology Centre, University of Torino, Torino, Italy; ^3^2i3T Società per la Gestione dell'Incubatore di Imprese e per il Trasferimento Tecnologico Scarl, University of Torino, Torino, Italy; ^4^Cell Factory of the University of Torino, Torino, Italy; ^5^Unicyte Srl, Torino, Italy

## Abstract

Cell therapy may be regarded as a feasible alternative to whole organ transplantation to treat end-stage liver diseases. Human liver stem cells (HLSCs) are a population of cells easily obtainable and expandable from a human adult liver biopsy. HLSCs share with mesenchymal stromal cells the same phenotype, gene expression profile, and differentiation capabilities. In addition, HLSCs show a specific commitment to the hepatic phenotype. Injection of HLSCs into immunodeficient mice fed with a methionine-choline-deficient diet to induce nonalcoholic steatohepatitis ameliorates liver function and morphology. In particular, HLSC treatment induced a reduction of liver fibrosis and inflammation at morphological and molecular levels. Moreover, HLSCs were able to persist for up to 3 weeks after the injection. In conclusion, HLSCs have healing effects in a model of chronic liver disease.

## 1. Introduction

Nonalcoholic fatty liver disease is a cause of chronic liver disease worldwide. A fraction of patients with nonalcoholic fatty liver disease (about 30%) develop nonalcoholic steatohepatitis (NASH), characterized by liver fibrosis and inflammation eventually leading to cirrhosis, hepatocellular carcinoma, and other liver-related complications. The prevalence of NASH in Western countries with nonalcoholic fatty liver disease is 20-30% and is steadily increasing in parallel with the rising prevalence of obesity and insulin resistance [1, 2].

Liver transplantation is currently the standard therapy for end-stage liver disease including NASH. In addition, cirrhosis leading to NASH is the second most common indication for liver transplantation in the USA. However, high cost, lifelong immunosuppressive agents, and a shortage of organ donors limit the availability of such treatment [2, 3]. Lately, cell-based therapy is emerging as a possible alternative for the treatment of NASH manifestations. In particular, bone marrow-derived mesenchymal stromal cells (MSCs), which have exhibited therapeutic efficacy in experimental models of NASH [4], show functional engraftment of transplanted cells [5, 6]. In addition, hepatocytes derived by differentiating induced pluripotent stem cells have been successfully used for the treatment of NASH in mice [7].

Herrera et al. isolated and characterized a population of multipotent stem cells from a human liver biopsy which exhibited a high proliferative capacity [8]. This population of cells currently known as human liver stem cells (HLSCs) express many surface markers in common with MSCs (CD73, CD29, CD105, CD90, and CD44), various stem cell and embryonic markers (Nanog, Oct3/4, Sox2, Musashi, SSEA4, and Pax2), and markers specific to hepatic cells (albumin, cytokeratin-8, and cytokeratin-18) [8]. HLSCs, similar to MSCs, also exhibit immunomodulatory properties [9]. Furthermore, they are able to differentiate into osteocytes, endothelial cells, and functional hepatocytes, as well as form islet-like structures [8, 10, 11]. When injected in different experimental models of acute liver injury, HLSCs have not only improved liver function and morphology but also engrafted into injured livers [8, 12]. Moreover, HLSCs are also able to improve the recovery of injury in other organs, such as the kidneys [13]. In this work, we evaluated whether HLSCs, obtained and expanded in Good Manufacturing Practice (GMP) conditions, may offer an alternative option for the treatment of NASH-associated fibrosis and inflammation.

## 2. Materials and Methods

### 2.1. Isolation and Culture of HLSCs in GMP Conditions

A HLSC-master cell bank (MCB) was generated by Anemocyte International S.v.I. (Gerenzano, Italy) from a 10-15 mm liver fragment obtained from a liver donor, according to standard criteria of Centro Nazionale Trapianti, as described [8]. The liver fragment was digested in Good Manufacturing Practice- (GMP-) grade collagenase (NBI, 0.6 mg/ml) and neutral protease (NBI, 0.73 mg/ml) for 30 minutes at 37°C. The liver cell suspension, obtained from enzymatic digestion, was washed (400 g for 10 minutes) and cultured (2.5 × 10^5^/ml in a T75 flask with a 10 ml/flask) in the presence of a GMP medium, composed of minimal essential medium (*α*-MEM, Lonza, Basel, Switzerland) supplemented with 10% fetal calf serum (Gibco, Cambrex), 10 ng/ml of human recombinant epidermal growth factor (Miltenyi Biotec, Bergisch Gladbach, Germany), 10 ng/ml of human recombinant fibroblast growth factor basic (Miltenyi Biotec, Bergisch Gladbach, Germany), 2 nM of L-glutamine (Lonza), and 100 U/ml of penicillin/streptomycin (from Sigma-Aldrich, St. Louis, MO, USA) and maintained in a humidified 5% CO_2_ incubator at 37°C. HLSCs, obtained from the liver cell suspension after 2 weeks of culture, were seeded at the density of 2.5 × 10^5^ cells per flask (T75) in the same culture medium. Once the cells reached up to about 80% of confluency, they were harvested and centrifuged at 400 *g* for 5 minutes and used for experimental purposes. All reagents used were suitable for clinical use and produced in conditions analogous to European and Italian GMP.

Routine characterization of HLSCs was performed by cytofluorimetric analyses using the Guava easyCyte Flow Cytometer (Millipore, Billerica, MA, USA) and analysed with InCyte software for the expression of typical MSC (CD29, CD73, and CD105 all from Miltenyi Biotec) and hepatic (Albumin, LSBio, LifeSpan BioSciences, Seattle, Washington) markers. Indirect immunofluorescence was performed on HLSCs cultured on chamber slides (Nalge Nunc International, Rochester, NY), fixed in 4% paraformaldehyde containing 2% sucrose, and permeabilized with HEPES Triton X-100 buffer. The following monoclonal antibodies (Ab) were used: anti-*α*-fetoprotein, anti-albumin (R&D Systems, Abington, UK), anti-*α*-smooth muscle actin (*α*-SMA) (Dako Denmark A/S), anti-cytokeratin-18 (Chemicon International, Temecula, CA,), anti-cytokeratin-8, and anti-cytokeratin-19 (Santa Cruz Biotechnology, CA, USA). Anti-vimentin (Sigma-Aldrich), anti-nestin, anti-Musashi, anti-Nanog, anti-Oct3/4, anti-SSEA4, and anti-Sox2 rabbit polyclonal antibodies (Abcam, Cambridge, UK) were used. Omission of the primary antibodies was used as the control where appropriate. Alexa Fluor 488 or Texas Red anti-rabbit IgG or anti-mouse IgG (Molecular Probes, Leiden, Netherlands) was used as secondary Ab. Confocal microscopy analysis was performed using a Zeiss LSM 5 Pascal Model Confocal Microscope (Zeiss International, Jena, Germany). Hoechst 33258 dye (Sigma-Aldrich) was added for nuclear staining.

### 2.2. Culture of MSCs

Bone marrow MSCs were obtained from Lonza and cultured and characterized as previously described [14]. The cell preparations used were positive for the typical MSC markers (CD105, CD29, CD73, CD44, and CD90) and had the ability to differentiate into adipogenic and osteogenic lineages (not shown).

### 2.3. Telomere Length

DNA from HLSCs at different passages (6 to 17 passages) was extracted with the DNeasy Blood & Tissue Kit (QIAGEN) according to the manufacturer's protocol. Telomere length was evaluated by real-time PCR with the Absolute Human Telomere Length Quantification qPCR Assay Kit (ScienCell Research Laboratories, Carlsbad, CA, USA) following the manufacturer's protocol.

### 2.4. Human Mesenchymal Stem Cell PCR array

RNA was extracted by TRIzol (Ambion) from MSCs (Lonza) and HLSCs at different culture passages. Samples were retrotranscribed with the RT^2^ First Strand Kit and gene expression was analysed by RT^2^ Profiler™ PCR Array Human Mesenchymal Stem Cells (PAHS-082Z, QIAGEN) following the manufacturer's protocol.

### 2.5. *In Vivo* Model

Animal studies were conducted in accordance with the National Institutes of Health Guide for the Care and Use of Laboratory Animals. All procedures were approved by the Italian Health Ministry (authorization number: 419/2016-PR).

NASH was induced by continuous feeding of mice with methionine-choline-deficient diet (MCDD), as previously reported [5, 7]. MCDD induces fast, reproducible, and severe liver damage. Moreover, MCDD activates similar mechanisms of fibrosis and inflammation as in human NASH progression and so is ideal for studying strategies to inhibit these processes [15].

**Figure 1:**
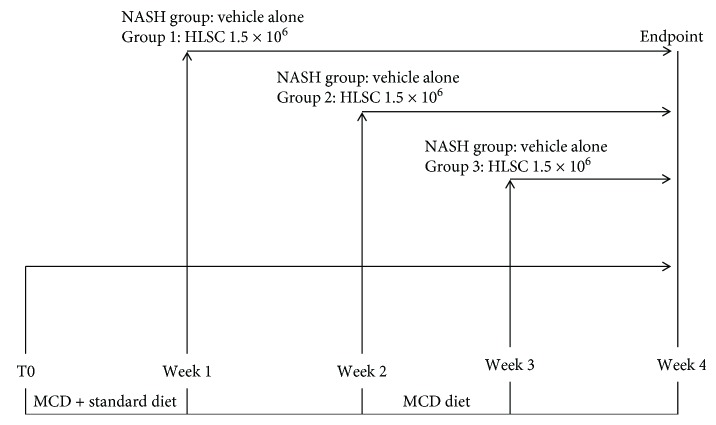
Schematic overview of the experimental design to test HLSCs in MCDD-fed mice, showing the week of MCDD and HLSCs or vehicle administration.

Severe Combined Immunodeficient (SCID, Charles River Laboratories, Wilmington, MA, USA) male mice, 10 weeks old, were accustomed to a MCDD (MP Biomedicals, Eschwege, Germany) by feeding a mixture of standard and MCD chow for 1 week. Thereafter, the full MCD chow was given. HLSCs (1.5 × 10^6^) from MCB, at passages 7 to 9 of cultures, were injected intravenously (tail vein) at different time points after the beginning of MCDD (Figure 1), at weeks 1 (Group 1: *n* = 11), 2 (Group 2: *n* = 9), or 3 (Group 3: *n* = 9). The NASH control group was injected with the vehicle alone (PBS) (*n* = 5/week). Control animals (*n* = 12) were fed with standard diet. For the dose/response study, NASH mice were injected at week 2 with different doses of HLSCs (*n* = 6 for 0.5 × 10^6^ and *n* = 8 for 3.0 × 10^6^). All animals were sacrificed at week 4, and the blood and liver were recovered for biochemical, histological, and molecular analyses.

### 2.6. Histological Analyses

Liver morphology was evaluated through formalin-fixed paraffin-embedded tissue staining. Briefly, 5 *μ*m thick paraffin liver sections were routinely stained, for microscopic evaluation, with hematoxylin and eosin (H&E, Merck) or Sirius Red for collagen.

Liver fibrosis was quantified by measuring collagenous fibrotic areas stained in red (sections stained with Sirius Red) in 10 random fields/section from images taken at a magnification of 400x, using multiphase image analyses with ImageJ software version 1.49s [16]. The surface area occupied by steatosis vacuoles was quantified in 10 random fields/section from images taken at a magnification of 200x, using multiphase image analyses with ImageJ software. Immunohistochemistry for the detection of proliferating hepatocytes was performed on paraffin liver sections. Briefly, paraffin sections were subjected to antigen retrieval, followed by blocking and labelling with 1 : 400 of anti-PCNA (Santa Cruz Biotechnology). Immunoperoxidase staining was then performed using a 1 : 300 dilution of the secondary HRP antibody (Pierce). Scoring for PCNA-positive cells was carried out by counting the number of positive nuclei per HPF (high-power field, 40x) in 10 randomly chosen sections.

Immunofluorescence was performed on 5 *μ*m thick cryostat sections. Sections were stained with mouse anti-CD45 (Biorbyt, San Francisco, CA, USA), mouse anti-S100A4 (fibroblast-specific protein 1 (FSP1), Abcam, Cambridge, MA, USA), or rabbit anti-human leukocyte antigen (HLA) class I rabbit (Santa Cruz Biotechnology) antibodies for 2 hours at 4°C. Rabbit anti-mouse FITC or anti-rabbit FITC (Molecular Probes) was used as secondary antibodies. Hoechst 33258 dye (Sigma-Aldrich) was added for nuclear staining. Confocal microscopy analysis was performed using a Zeiss LSM 5 Pascal Model Confocal Microscope (Zeiss International).

### 2.7. Quantitative Real-Time PCR

Total RNA was extracted from the liver tissue of the control or NASH mice treated with or without HLSCs using TRIzol™ reagent (Ambion, Thermo Fisher) according to the product's instruction. Briefly, mouse hepatic tissue was resuspended in 1 ml of TRIzol™ solution and homogenised in a Bullet Blender instrument (Next Advance Inc., New York, NY, USA) at a speed of 8 for 3 min using 0.5 mm size zirconium oxide beads and centrifuged at 12,000 *g* for 15 min at 4°C. Supernatant from homogenised tissue was used to isolate RNA, as mentioned above, which was quantified spectrophotometrically (NanoDrop ND-2000, Thermo Fisher Scientific). For gene expression analysis, quantitative real-time PCR was performed as described previously [17]. Briefly, first-strand cDNA was synthesised from 200 ng of total RNA using the High-Capacity cDNA Reverse Transcription Kit (Applied Biosystems, Foster City, CA). qRT-PCR was performed using the StepOnePlus RT-PCR machine (Applied Biosystems, Foster City, CA) in a 20 *μ*l reaction mixture containing 5 ng or 10 ng of cDNA template (according to tested gene expression analysis), the sequence-specific oligonucleotide primers (purchased from MWG Biotech, Eurofins Scientific, Brussels, Belgium), and the Power SYBR Green PCR Master Mix (Applied Biosystems). The *GAPDH* or *TBP* gene was used as a housekeeping gene to normalize RNA inputs. Fold change expressions with respect to control were calculated for all samples using the ΔΔCt method. The primers used for qRT-PCR are reported in Table 1.

### 2.8. Whole Genome DNA Analysis

Whole genome DNA was extracted from the frozen tissues of MCDD-fed mice treated or not with HLSCs at different time points using a DNA isolation kit (QIAGEN). Following quantification, 200 ng of DNA was amplified through PCR for human-specific DNA repeat *α*-satellite chromosome 17 (1171 bp fragment) (hu-*α*-sat-Ch-17) using the following primers: forward 5′-ACACTCTTTTTGCAGGATCTA-3′ and reverse 5′-AGCAATGTGAAACTCTGGGA-3′ [18]. The PCR samples were then analysed using agarose gel electrophoresis and analysed using the Gel Doc system (Bio-Rad). DNA isolated from HLSCs was used as a positive control and water as a negative control.

### 2.9. Statistical Analysis

Data analyses were performed using GraphPad Prism 6.0. Results are expressed as mean ± SD. Statistical analyses were performed by employing analysis of variance (ANOVA) with the Newman–Keuls test. A *p* value of <0.05 was considered significant.

## 3. Results

### 3.1. Characterization of HLSCs Obtained and Cultivated in GMP Conditions

**Figure 2:**
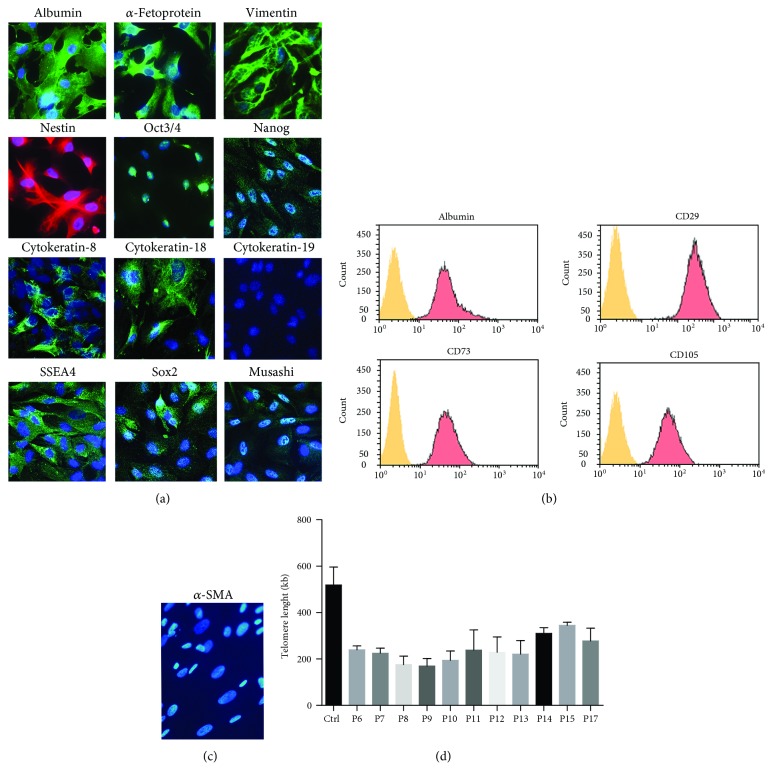
Characterization of HLSC-master cell bank. (a, c) Representative confocal micrographs showing the expression of several hepatic, mesenchymal, and embryonic stem cell markers in HLSCs (original magnification at ×400). Three experiments were performed with similar results of HLSCs at passage 6 of the culture. (b) Representative FACS analyses of HLSCs showing the expression of albumin, CD29, CD73, and CD105 (red histogram). Yellow histograms represent isotypic control. Three experiments were performed with similar results of HLSCs at passage 6 of the culture. (d) Telomere length of HLSCs was measured using the Absolute Human Telomere Length Quantification qPCR Assay Kit. A single copy reference (SCR) primer set recognizes and amplifies a 100 bp long region on human chromosome 17 and serves as reference for data normalization. The reference genomic DNA sample with known telomere length (Ctrl) serves as a reference for calculating the telomere length of MCB.

The HLSC-master cell bank was characterized as previously reported [8]. Immunocytochemistry revealed HLSCs to be 100% positive for the hepatocyte precursor marker *α*-fetoprotein, mature hepatocyte protein (albumin), the stem cell markers vimentin and nestin, and embryonic stem cell markers (Oct3/4, Nanog, SSEA4, Sox2, and Musashi1). HLSCs were also positive for cytokeratin-8 and cytokeratin-18 at about 20-30% (Figure 2(a)) and negative for the hepatic oval cell marker cytokeratin-19. HLSCs did not express *α-Sma* (Figure 2(c)). Flow cytometric analysis revealed that HLSCs were positive for several MSC markers such as CD29, CD73, and CD105 and further confirmed the expression of albumin (Figure 2(b)).

Telomere length is a marker of cellular senescence [19]. For this reason, we evaluated the telomere length during expansion of HLSCs from P6 to P17 (Figure 2(d)). Despite an initial reduction, HLSC telomere length remained stable from P7 to P17, with a mean value of 239 ± 57 kb, indicating that HLSCs remained stable for over seventeen culture passages.

**Figure 3:**
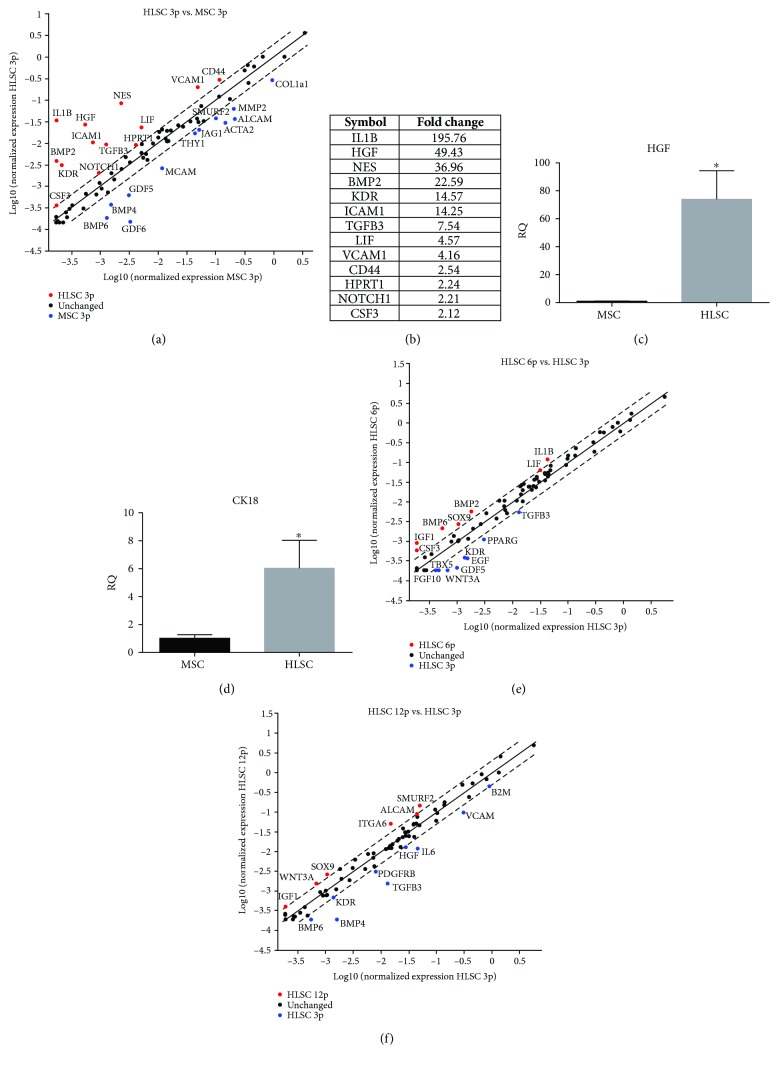
Gene expression profile of HLSCs. (a) Scatter plot showing the comparison of gene expression, screened by human MSC PCR array (PAHS-082Z) which includes genes that define the stemness, pluripotency, and self-renewal characteristics of MSCs and HLSCs at passage 3. The array does not include epithelial markers. Red dots represent genes more expressed by HLSCs, while blue dots represent genes more expressed by MSCs. Black dots are genes not differentially expressed. (b) Relative quantification (RQ) of genes more expressed by HLSCs with respect to MSCs. (c, d) Real-time PCR of hepatocyte growth factor (HGF) and cytokeratin-18 (CK18) expression in HLSCs compared with MSCs. Data are expressed as relative quantification using the ΔΔCt method. Normalization was made using TBP as a housekeeping gene. ANOVA with the Newman-Keuls multicomparison test was performed; ^∗^*p* < 0.05 HLSCs *versus* MSCs. (e, f) Scatter plots comparing the gene expression of HLSCs at passage 3 *versus* passage 6 (e) and passage 12 (f). Red dots represent genes more expressed by HLSCs at passage 6 or 12, while blue dots represent genes more expressed by HLSCs at passage 3. Black dots are genes not differentially expressed.

Since HLSCs and MSCs share the same phenotype, differentiation, and immunomodulatory properties, we decided to compare the gene expression profile of HLSCs with that of bone marrow-derived MSCs, using a specific MSC PCR array. The expression of 84 key genes involved in self-renewal and specific-MSC-differentiation capacities was evaluated. HLSCs showed a very similar gene expression profile to MSCs, on comparing the two cell populations at passage 3. Only 13 genes, out of 84, were differentially expressed in HLSCs with respect to MSCs (Figures 3(a) and 3(b)). In addition, the gene expression profile confirmed the presence of specific hepatic markers, such as hepatocyte growth factor (HGF) (Figures 3(a)–3(c)) and cytokeratin-18 (Figure 3(d)) in HLSCs. Moreover, a comparison between HLSCs at passage 3 of the culture and HLSCs at passages 6 and 12 revealed a similarity between the gene expression profile up to 12 culture passages, indicating the stability of HLSCs *in vitro* (Figures 3(e) and 3(f)).

### 3.2. HLSCs Improve Liver Function and Morphology

**Figure 4:**
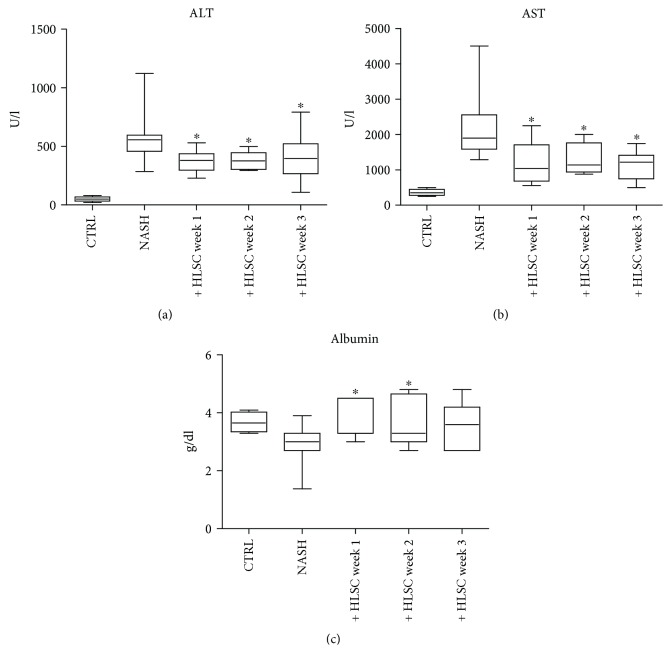
Effect of HLSC injection on liver function of MCDD-fed mice. Aspartate aminotransferase (AST) (a), alanine aminotransferase (ALT) (b) expressed as U/l, and albumin (c) expressed as g/dl were measured as biomarkers of liver cell injury in serum of control SCID mice (CTRL), in mice fed with MCDD for 4 weeks and injected with vehicle alone (NASH), and in MCDD-fed mice treated with intravenous injection of 1.5 × 10^6^ HLSCs at week 1 (Group 1, *n* = 11), 2 (Group 2, *n* = 9), or 3 (Group 3, *n* = 9) and sacrificed at week 4. Data are expressed as mean ± standard deviation (SD). ANOVA with Newman-Keuls multicomparison test was performed; ^∗^*p* < 0.05 HLSC-treated mice *versus* NASH mice.

To evaluate the potential therapeutic ability of HLSCs in a mouse model of NASH, we induced NASH by feeding SCID mice with a MCDD for 4 weeks. During this period, we assessed the efficacy of HLSC administration at different time points (Figure 1). Mice on a MCDD had an increase of alanine aminotransferase (ALT) and aspartate aminotransferase (AST) in the plasma, while albumin levels decreased (Figure 4). However, on injecting HLSCs at different time points of MCDD, a significant decrease in the plasma levels of ALT and AST was observed (Figure 4). In addition, serum albumin levels were restored to healthy control levels by HLSCs injected at weeks 1 and 2 (Figure 4).

**Figure 5:**
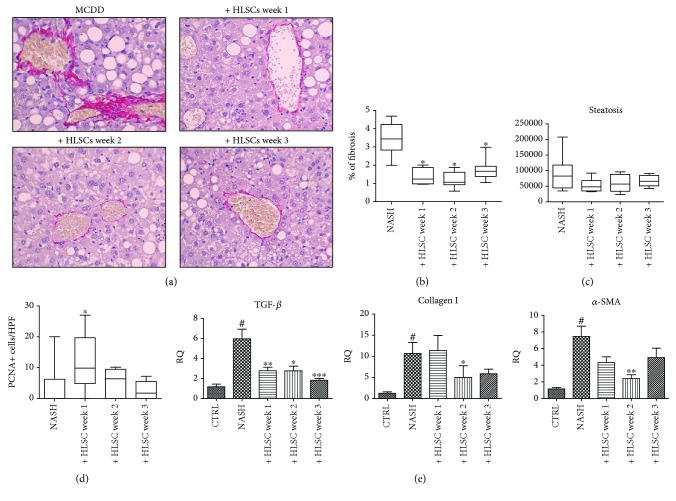
Effects of HLSCs on liver morphology and fibrosis. (a) Representative light microscopy micrographs of liver histology of mice at week 4 of MCDD, treated with intravenous injection of 1.5 × 10^6^ HLSCs or with vehicle alone (NASH) (original magnification 400x). Sirius Red staining showed fibrosis in the perivenous area of MCDD-fed mice. Red stain represents collagen fibers considered to be a marker of liver fibrosis. (b) Histological quantification of fibrosis in MCDD-fed mice injected with HLSCs or with vehicle alone (NASH) at different weeks, by multiphase image analyses of 10 fields per section (original magnification 400x). Data shown represents mean ± SD. ANOVA with Newman-Keuls multicomparison test was performed: ^∗^*p* < 0.05 MCDD-fed mice injected with 1.5 × 10^6^ HLSCs at different weeks (1 *n* = 11, 2 *n* = 9, or 3 *n* = 9) *versus* MCDD-fed mice injected with vehicle alone (NASH). (c) Histological quantification of the surface area occupied by steatosis vacuoles in MCDD-fed mice injected with HLSCs or with vehicle alone (NASH) at different weeks (1 *n* = 11, 2 *n* = 9, or 3 *n* = 9) by multiphase image analyses of 10 fields per section (original magnification 200x). Data shown represents mean ± SD. (d) Quantification of PCNA-positive cells/high-power field (HPF) (original magnification ×400) in 10 fields per section. Data represents mean ± SD. ANOVA with the Newman-Keuls multicomparison test was performed: ^∗^*p* < 0.05 MCDD-fed mice injected with 1.5 × 10^6^ HLSCs at different weeks (1 *n* = 11, 2 *n* = 9, or 3 *n* = 9) *versus* MCDD-fed mice injected with vehicle alone (NASH). (e) Gene expression levels of fibrotic markers *Tgf-β*, *Col 1a1*, and *α-Sma* in livers of MCDD-fed mice treated with vehicle alone or with HLSCs (1.5 × 10^6^) at different weeks (1 *n* = 11, 2 *n* = 9, or 3 *n* = 9). Data are expressed as relative quantification using the ΔΔCt method. Normalization was made using GAPDH as a housekeeping gene. ANOVA with the Newman-Keuls multicomparison test was performed; ^∗^*p* < 0.05, ^∗∗^*p* < 0.01, and ^∗∗∗^*p* < 0.001 HLSC-treated mice *versus* NASH mice treated with vehicle alone; ^#^*p* < 0.05 MCDD-fed mice NASH treated with vehicle alone *versus* control animals fed with standard diet.

One of the main hallmarks of NASH is liver fibrosis. Histologically, fibrosis was highlighted through Sirius Red staining of collagen, which was increased in MCDD-fed mice, primarily in both perivenous and periportal areas of the liver (Figure 5(a)). Compared to vehicle-treated animals, MCDD-fed mice that received HLSCs at week 1, 2, or 3 showed a significant reduction in fibrosis (Figures 5(a) and 5(b)). Collagen is secreted by activated stellate cells, which are characterized by the expression of *α*-SMA [20]. mRNA expression levels of *Col1a1* (collagen I) and *α-Sma* were elevated in the livers of MCDD-fed mice, compared to mice fed with the standard diet (Figure 5(e)). The mRNA levels of *α-Sma* and *Col1a1* decreased in NASH mice treated with HLSCs at different time points with a statistically significant reduction observed in NASH mice injected at week 2 (Figure 5(e)). Moreover, the mRNA expression level of the fibrogenic factor *Tgf-β* was significantly reduced in MCDD-fed mice injected with HLSCs at the different time points tested (Figure 5(e)).

Transplantation of HLSC also attenuated steatosis, but the reduction did not reach statistical significance (Figure 5(c)). Moreover, an injection of HLSCs at week 1 of MCDD induced an increase in the proliferation of hepatocytes as inferred by PCNA staining (Figure 5(d)).

**Figure 6:**
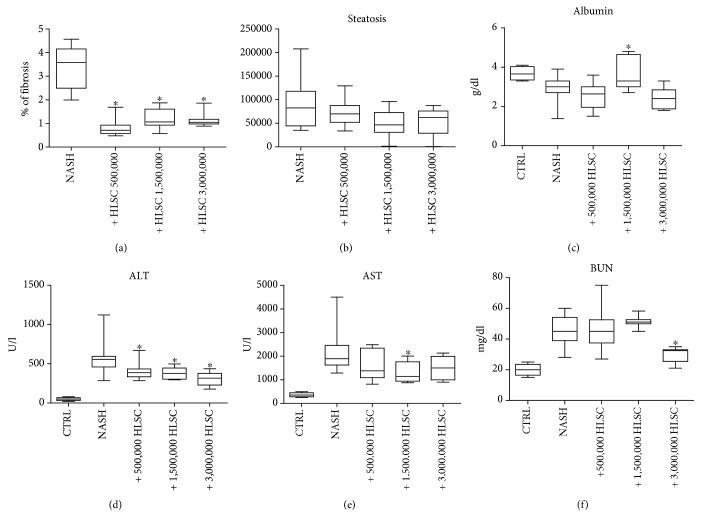
Effects of different amounts of HLSCs on liver function and morphology in MCDD-fed mice. (a) Histological quantification of fibrosis in MCDD-fed mice injected with different amounts of HLSCs (0.5, 1.5, or 3 × 10^6^ and *n* = 6, 9, and 8, respectively) or with vehicle alone at week 2, by multiphase image analyses of 10 fields per section (original magnification 400x). Data represents mean ± SD. ANOVA with the Newman-Keuls multicomparison test was performed: ^∗^*p* < 0.05 MCDD-fed mice injected with different doses of HLSCs *versus* MCDD-fed mice injected with vehicle alone (NASH). (b) Histological quantification of the surface area occupied by steatosis vacuoles in MCDD-fed mice injected with different amounts of HLSCs (0.5, 1.5, or 3 × 10^6^ and *n* = 6, 9, and 8, respectively) or with vehicle alone at week 2, by multiphase image analyses of 10 fields per section (original magnification 200x). Data represents mean ± SD. Albumin (c), alanine aminotransferase (ALT) (d), aspartate aminotransferase (AST) (e), and blood urea nitrogen (BUN) (f) were measured in the serum of control SCID mice (CTRL), in mice fed with MCDD for 4 weeks and injected with vehicle alone (NASH), and in MCDD-fed mice treated with intravenous injection of different amounts of HLSCs (0.5, 1.5, or 3 × 10^6^ and *n* = 6, 9, and 8, respectively) at week 2 and sacrificed at week 4. Data are expressed as mean ± SD. ANOVA with the Newman-Keuls multicomparison test was performed; ^∗^*p* < 0.05 HLSC-treated mice *versus* NASH mice treated with vehicle alone.

To define the optimal dose of HLSCs to obtain a significant improvement in liver function and morphology, a dose/response study was performed whereby different concentrations of HLSCs were injected at week 2 of MCDD. All the cell doses tested (0.5, 1.5, and 3.0 × 10^6^) induced a reduction in fibrosis (Figure 6(a)). Furthermore, serum markers of the liver function indicated that all the cell doses tested showed significantly normalized ALT levels. However, AST and albumin levels were improved significantly only with the dose of 1.5 × 10^6^ cells (Figures 6(c)–6(e)). In the case of blood urea nitrogen levels in the plasma, only the higher dose tested induced a significant reduction (Figure 6(f)).

### 3.3. HLSCs Reduce Liver Inflammation

**Figure 7:**
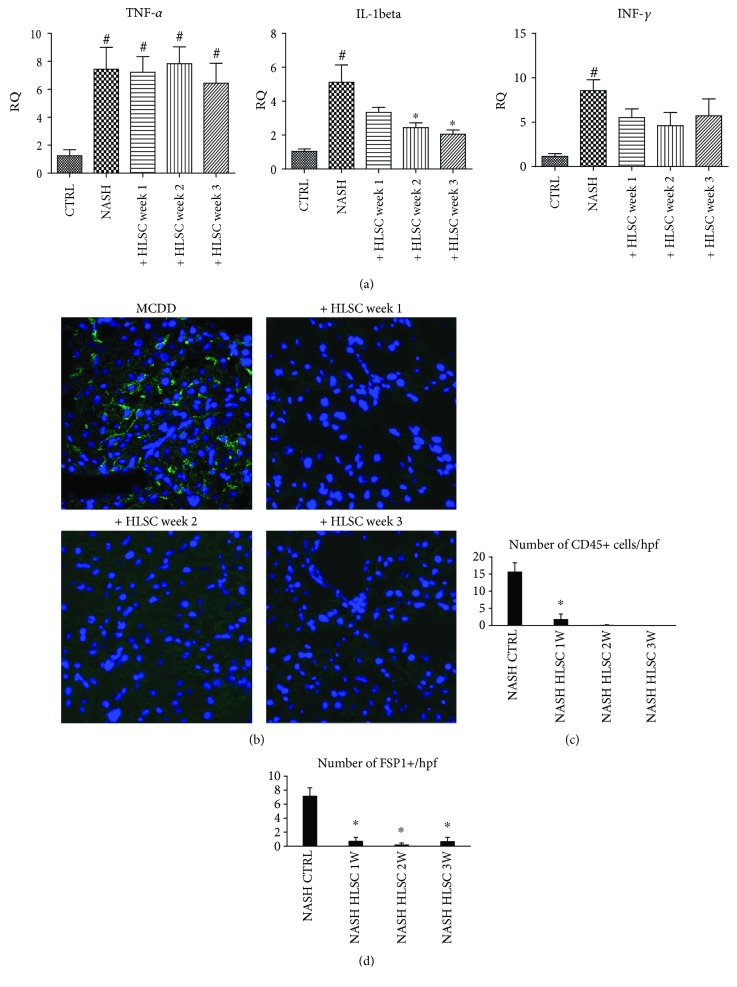
Effects of HLSCs on liver inflammation. (a) Gene expression levels of proinflammatory cytokines *Tnf-α*, *Il-1β*, and *Ifn-γ* in livers of MCDD-fed mice treated at different weeks (1 *n* = 11, 2 *n* = 9, or 3 *n* = 9) with 1.5 × 10^6^ HLSCs or with vehicle alone. Data are expressed as relative quantification using the ΔΔCt method. Normalization was made using GAPDH as a housekeeping gene. ANOVA with the Newman-Keuls multicomparison test was performed; ^∗^*p* < 0.05 HLSC-treated mice *versus* NASH mice treated with vehicle alone and ^#^*p* < 0.05 MCDD-fed mice *versus* CTRL mice treated with standard diet. (b) Representative micrographs of liver cryosections from MCDD-fed mice injected or not with HLSCs at week 1, 2, or 3, stained for CD45 to identify the presence of inflammatory cells. Infiltrates of inflammatory cells. CD45-positive cells (green) were present only in MCDD-fed mice that did not receive cell treatment. Nuclei were counter-stained with DAPI (blue). Original magnification 400x. Quantification of CD45-positive cells (c) and of FSP1-positive cells (d) per HPF (original magnification ×400) in 10 fields per section. Data represents mean ± SD. ANOVA with the Newman-Keuls multicomparison test was performed: ^∗^*p* < 0.05 MCDD-fed mice (NASH) treated with vehicle alone *versus* MCDD-fed mice injected with 1.5 × 10^6^ HLSCs at different weeks.

Along with fibrosis, NASH is also associated with liver inflammation [4, 6]. Liver tissue of MCDD-fed mice displayed a significant upregulation in the mRNA levels of proinflammatory cytokines such as *Tnf-α*, *Il-1β,* and *Ifn-γ* (Figure 7(a)). However, injection with HLSCs induced a reduction in mRNA expression levels of both *Il-1β* and *Ifn-γ* indicating an anti-inflammatory effect of HLSCs (Figure 7(a)).

To complete the evaluation of the inflammatory status of the liver tissue of MCDD-fed mice injected with or without HLSCs, we evaluated the presence of inflammatory cells, using specific markers, such as CD45 (common leukocyte antigen) and FSP1 (fibroblast-specific protein 1), that recognize a specific subset of inflammatory macrophages during liver injury, fibrosis, and cancer [21]. CD45^+^ cells and/or FSP1^+^ cells were present in the livers of MCDD-fed mice. On the other hand, no inflammatory infiltrates were observed in mice injected with HLSCs at different time points (Figures 7(b) and 7(c)).

### 3.4. HLSC Engraftment

**Figure 8:**
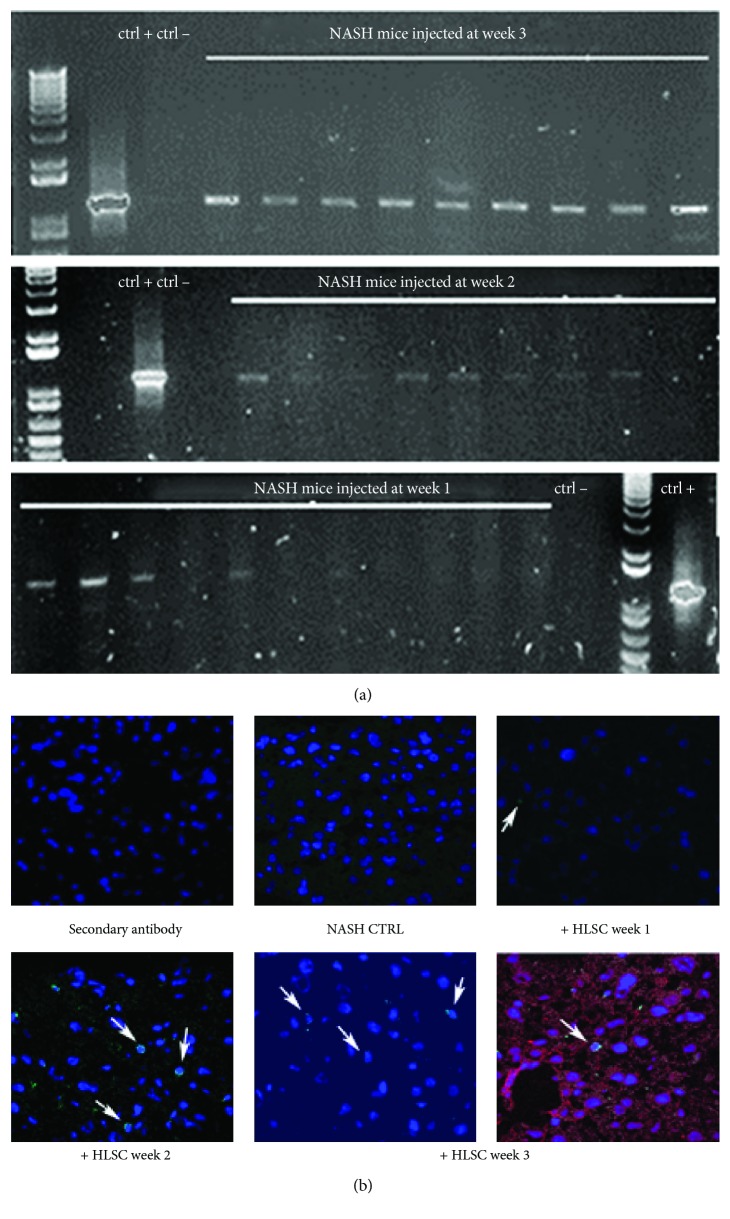
HLSC engraftment in MCDD-fed mice. (a) Whole genome DNA analysis (*α*-sat-ch-17) of mouse livers sacrificed at week 4 of MCDD and injected with HLSCs at week 1, 2, or 3. Ctrl+: positive control using HLSCs; Ctrl-: H_2_O. (b) Representative confocal micrographs showing the presence of human cells localized widely in the liver parenchyma of MCDD-fed mice injected with vehicle alone (NASH CTRL) or with 1.5 × 10^6^ HLSCs at week 1, 2, or 3, as evaluated by HLA-class I (green). Costaining of murine cytokeratin-8 (red) and HLA-class I (green) at week 3 is shown. Nuclei were counter-stained with DAPI (blue). Original magnification 400x. Arrows indicate HLA-class I-positive cells.

The presence of HLSCs (human cells) injected in MCDD-fed mice at different time points was evaluated through the analysis of hu-*α*-sat-Ch-17 in mouse DNA. We found that hu-*α*-sat-Ch-17 was detectable in all NASH (9 out of 9) mice injected with HLSCs at week 3 (sacrificed at week 4), in 6 out of 9 NASH mice injected with HLSCs at week 2 (sacrificed at week 4), and in 4 out of 11 NASH mice injected at week 1 (sacrificed at week 4) (Figure 8(a)). This therefore indicates the successful engraftment of HLSCs in treated mice for up to 3 weeks (Figure 8(a)). Immunofluorescence staining with an antibody against HLA-class I showed the presence of human cells in mice injected at different time points and sacrificed at week 4 (Figure 8(b)). HLA-1^+^ cells were observed to be widely spread in the hepatic tissue (Figure 8(b)). Furthermore, they did not coexpress markers of hepatic differentiation such as cytokeratin-8 and cytokeratin-18 or albumin (data not shown), suggesting that undifferentiated HLSCs persisted in the liver parenchyma. No HLSC localization was observed in healthy mice injected with cells (not shown) or in mice with liver damage that were injected with vehicle alone. This data therefore confirms the molecular analyses done above.

## 4. Discussion

Cell therapy is regarded as a feasible alternative to whole organ transplantation for the treatment of end-stage liver diseases. A shortage in donor organs for the isolation of human hepatocytes in sufficient quantity has increased the need to find alternative cell sources such as stem/progenitor cells. HLSCs are a stem cell population that are easily obtainable from human adult liver biopsies [8]. HLSCs express many surface markers in common with hMSCs and embryonic and hepatic cells. Moreover, HLSCs were negative for the expression of cytokeratin-19, a marker of oval cells, and for alpha-SMA, a marker of activated stellate cells. At variance, adult-derived human liver mesenchymal-like cells described by Najimi et al. [22] expressed *α-Sma* and cytokeratin-19, have CyP3A4 activity in the basal condition, and secrete urea suggesting a more differentiated stage in respect to HLSCs, which expressed CyP3A4 activity and secrete urea only after differentiation in hypogravity condition [8, 10]. HLSCs in an undifferentiated state have a great potential to proliferate and remain stable for over seventeen culture passages, as demonstrated by telomere length and gene expression stability. Furthermore, we have confirmed that HLSCs exhibit a similar phenotype and gene expression profile to MSCs. At the same time, they also maintain a specific commitment towards a hepatic phenotype. In addition, GMP protocols in order to obtain and expand HLSCs have been established in our laboratory. Moreover, they have been approved by the Italian Regulatory Agency for a phase I study on urea cycle disorders (phase 1 study EudraCT: 2012-002120-33).

In the present study, we have shown that HLSCs may exhibit a therapeutic potential in a MCDD-fed mouse model of NASH. Mice maintained in MCDD developed steatosis, accompanied by fibrosis and inflammation, which are considered the main features of NASH. Administration of HLSCs ameliorated fibrosis and inflammation as observed through histology and molecular analyses. In addition, markers of liver function, such as ALT, AST, and plasma albumin levels, were also restored to near normal levels. Different time points of HLSC injection in NASH mice have been tested, showing that early (week 1) and late (week 2 and 3) points of HLSC administration induced a significant improvement of liver function and morphology, at histological and molecular levels. Moreover, we tested different doses of HLSCs, injected at week 2. All the cell doses tested induced an amelioration of liver morphology, in particular of liver fibrosis. In regard to liver function, injection of 1.5 × 10^6^ HLSCs restored also serum albumin and only injection of a higher dose (3 × 10^6^ HLSCs) allowed an improvement of the BUN level.

Notably, as HLSCs are committed to a hepatic lineage, they do not require *in vitro* differentiation steps like bone marrow MSC and induced pluripotent stem cells in order to obtain beneficial effects in MCDD-fed mice [5–7].

To determine whether the improvement of NASH observed post-HLSC administration was related to the recruitment and engraftment of donor cells into the liver of the recipients, we evaluated the presence of human cells in HLSC-treated livers of mice, through histological and molecular analyses. Our data showed that human cells persisted up to 3 weeks in the liver of MCDD-fed mice. We have previously shown that HLSCs are able to engraft and contribute to hepatic regeneration in an acute liver injury model induced by *N*-acetyl-*p*-aminophenol [8]. Moreover, in a lethal model of liver injury, induced by D-galactosamine and lipopolysaccharide, HLSCs increased survival and were engrafted in the liver parenchyma. Furthermore, a small population of HLSCs coexpressed cytokeratin-8 and cytokeratin-18, seven days after injection, indicating a differentiation of HLSCs into hepatocytes [12]. Nonetheless, after 21 days, an undifferentiated population of HLSCs still persisted [12]. In the present study, several human cells detected in the liver parenchyma of MCDD-fed mice lacked the expression of specific markers of hepatic differentiation, suggesting the persistence of undifferentiated HLSCs. These data therefore indicate that differentiation of HLSCs into mature hepatocytes was not necessary to attenuate liver fibrosis and inflammation in this model.

A key factor in NASH is inflammation characterized by the infiltration of inflammatory cells and increased expression of proinflammatory cytokines in the hepatic tissue. Treatment with HLSCs alleviated liver inflammation in MCDD-fed mice, through the reduction in levels of proinflammatory cytokines, as has also been described in different animal models, whereby the local downregulation of proinflammatory cytokines is associated with tissue protection and regeneration [23, 24]. Furthermore, the reduction in leukocyte infiltration observed in MCDD-fed mice treated with HLSCs could explain the reduction in fibrosis development, since infiltrating macrophages play a pivotal role in the fibrogenic process [25–27].

A hallmark of NASH-associated fibrosis is the activation of hepatic stellate cells that is characterized by an increase in *α*-SMA expression and collagen I deposition [28]. The levels of both markers were significantly increased in the liver of MCDD-fed mice and returned to near normal levels posttreatment with HLSCs. These data therefore indicate that the injection of HLSCs attenuated hepatic stellate cell activation. HLSCs could therefore influence directly the activation state of stellate cells or indirectly through the reduction of the hepatic inflammatory state. Moreover, TGF-*β*1 is considered a major driver of fibrosis [29] and plays a pivotal role in hepatic stellate cell activation [30, 31]. A remarkable achievement of our study is that HLSCs injected at different time points significantly reduced the expression level of TGF-*β* confirming the antifibrotic action of HLSCs.

HLSCs exerted their beneficial effect, at least in part, by reducing the expression of profibrogenic factors, as has also been shown for other stem cell types in different models of liver fibrosis [31–35]. The hepatic growth factor (HGF) is a well-known antifibrotic factor, and its role to ameliorate fibrosis and inflammation has been reported in various experimental models of NASH [36–38]. Furthermore, deletion of the HGF-receptor c-met leads to the development of severe NASH in mice [38]. Molecular analyses of HLSCs revealed the expression of HGF mRNA in high levels. The beneficial effect of HLSCs in the lethal model of acute liver failure depended, at least in part, on HGF production [12]. We therefore speculate that in the MCDD-fed model of NASH, HGF could play a relevant role in the beneficial effects observed when administering HLSCs.

## 5. Conclusion

Taken together, these results demonstrated the healing properties of HLSCs in a model of chronic liver disease. In fact, treatment with HLSCs exhibited not only antifibrotic effects but also anti-inflammatory effects in a murine model of NASH. Furthermore, these effects observed could be attributed to the downregulation of profibrotic and proinflammatory genes.

## Figures and Tables

**Table 1:** RT-PCR-specific primers.

Gene	Sense (5′→3′)	Antisense (5′→3′)
*m-Collagen type I*	ATCTCCCTGGTGCTGATGGAC	ACCTTGTTTGCCAGGTTCAC
*m-TGF-beta1*	GCAACAATTCCTGGCGTTACC	CGAAAGCCCTGTATTCCGTCT
*m-Alpha-smooth muscle actin*	CATCTCCGAAGTCCAGCACA	GACGCACCACTGAACCCTAA
*m-IL-1beta*	CAACCAACAAGTGATATTCTCCATG	GATCCACACTCTCCAGCTGCA
*m-INF-gamma*	GAGCCAGATTATCTCTTTCATCC	GTTGTTGACCTCAAACTTGG
*m-TNF-alpha*	CATCTTCTCAAAATTCGAGTGACAA	TGGGAGTAGACAAGGTACAACCC
*m-GADPH*	TGTCAAGCTCATTTCCTGGTATGA	TACTCCTTGGAGGCCATGT
*HGF*	CACACAGGTATATTTGCTGGATGATAA	GGGACACAAGGTATAAATTGTTTTGG
*CK-18*	TGGTACTCTCCTCAATCTGTCG	CTCTGGATTGACTGTGGAAGT
*TBP*	TGTGCACAGGACCCAAGAGT	ATGTGCACAGGACCCAAGAGT

## Data Availability

All data are included in the manuscript.
